# Pharmacology and Toxicology of Opioids—Recent Advances and New Perspectives

**DOI:** 10.3390/ph18071055

**Published:** 2025-07-18

**Authors:** Joana Barbosa, Ricardo Jorge Dinis-Oliveira, Juliana Faria

**Affiliations:** 1 Associate Laboratory i4HB—Institute for Health and Bioeconomy, University Institute of Health Sciences—CESPU, 4585-116 Gandra, Portugal; 2 UCIBIO—Applied Molecular Biosciences Unit, Translational Toxicology Research Laboratory, University Institute of Health Sciences (1H-TOXRUN, IUCS-CESPU), 4585-116 Gandra, Portugal; 3 Department of Public Health and Forensic Sciences, and Medical Education, Faculty of Medicine, University of Porto, 4200-319 Porto, Portugal; 4 FOREN-Forensic Science Experts, Av. Dr. Mário Moutinho 33-A, 1400-136 Lisboa, Portugal

This Special Issue of *Pharmaceuticals* comprises one review and four original research articles that explore the multifaceted pharmacological and toxicological profiles of both classical and atypical opioids across different clinical contexts and biological models. Opioids are not considered a homogeneous pharmacological class but are instead a range of compounds that includes medicines and illegal drugs with varying mechanisms of action, therapeutic potentials, and toxicological effects. When it comes to opioid use, although countries like the United States of America have experienced a noticeable decline in prescription over recent decades as a result of public health efforts to reduce side effects, misuse, and abuse, opioids remain the gold standard for cancer pain treatment and are still widely prescribed due to their efficacy [1,2,3,4]. Furthermore, other regions around the world, including Europe, have experienced an increase in opioid prescription and/or consumption over the last few years [5,6,7,8,9,10]. Despite strategies to limit prescription rates, overdoses, and deaths, particularly those involving opioids like fentanyl, consumption rates have continued to increase [11,12]. This ongoing trend underscores the complexity of the opioid crisis and underlines the pressing need for further research in this field. In this context, there is growing interest in atypical opioids, which may offer effective analgesia with less severe side effects and a lower abuse potential. Cebranopadol, deuterated mitragynine, and ADL5859, which act as agonists of other opioid receptors in addition to or instead of µ-opioid receptors, are three such examples at different stages of development. These compounds hold the potential to minimize the typical adverse effects of opioids [13,14,15]. There is also renewed interest in biased opioid agonists, owing to their preference for activating G protein-mediated signaling over the β-arrestin pathway, downstream of µ-opioid receptor binding. G protein signaling is associated with pain relief, whereas the β-arrestin pathway has been linked to adverse effects such as constipation, respiratory depression, tolerance, and dependence [16,17,18]. Under this category, oliceridine and tegileridine have recently been approved for the treatment of acute and post-operative pain, respectively, whereas SR-14968 has shown an improved safety profile in animal studies [19,20,21]. In turn, methocinnamox is a long-lasting μ-antagonist still in preclinical development, designed to outlast naloxone and prevent opioid overdose relapse [22]. Collectively, these examples illustrate how workable and fertile the area of new opioid development is, and how likely it is that new opioid options will emerge on the market in the coming years.

In this regard, J. Barbosa and co-workers report, in an original study, the neurotoxic effects of tramadol and tapentadol, two atypical opioids, on the rat hippocampal formation, with a special focus on molecular and cellular modifications associated with neurobehavioral toxicity [Contribution 1]. Their results show significant molecular and cellular alterations in the hippocampus, which are related to oxidative stress, neuroinflammation, and neurodegeneration. Although generally considered safer than classic opioids, tramadol and tapentadol are already being associated with significant neurotoxicity and behavioral dysfunction, highlighting the need for caution when prescribing these drugs, particularly for chronic use, due to the potential neurological side effects [23,24,25].

J. Boyd and co-workers studied the cardiopulmonary consequences of fentanyl overdose in male SKH1 mice to explore the persistence of physiological alterations after fentanyl exposure [Contribution 2]. Their results showed cardiopulmonary dysregulation and eventual persistent toxicity, including prolonged disturbances in heart and lung function. These results are consistent with known clinical observations in humans, where fentanyl’s high potency and rapid onset increase the risk of fatal respiratory depression and cardiovascular collapse [26]. Their study emphasizes the high risk associated with fentanyl overdose and its potentially severe impact on the cardiopulmonary system, reinforcing public health concerns regarding the risks associated with the misuse of this synthetic opioid [12].

As previously discussed, opioids remain the gold standard for cancer pain management, with morphine being one of the most widely prescribed medications for pain associated with various types of cancer [3,27]. However, the impact of morphine on cancer cells may differ depending on tumor type, a question explored by M.-O. Parat and co-workers [Contribution 3]. In their in vitro study, the authors evaluated how morphine modulates the proliferation of twenty cancer cell lines derived from six different organs. The results demonstrate that morphine had no significant or organ-specific effect on cancer cell proliferation, with only one cell line showing a significant increase in growth. These findings highlight the complexity of opioid–cancer interactions and contribute to the ongoing debate regarding the potential for morphine to influence tumor progression differentially [28,29]. Nevertheless, morphine continues to be widely used in cancer pain management due to its proven efficacy and essential role in supporting patient quality of life in cancer care [30]. In this regard, existing guidelines recommend individualized opioid use in cancer management, emphasizing that current evidence does not support avoiding morphine due to concerns about cancer progression, especially when pain control is needed [30].

Dezocine, a biased opioid agonist, is increasingly recognized as a potential treatment for opioid addiction due to its rapid onset and offset, and its ability to antagonize κ-opioid receptors and increase dopamine signaling in reward-related brain regions. These properties enable dezocine to reduce withdrawal symptoms, prevent relapses, and attenuate addictive behaviors [16,17,18]. Furthermore, since dezocine combines opioid receptor activation with serotonin and norepinephrine reuptake inhibition, it has the potential to be used in the treatment of neuropathic and chronic pain, including cancer pain [31]. While it does have the potential to induce respiratory depression and euphoric effects, these outcomes are limited by a ceiling effect [18], which supports its application beyond the treatment of acute pain. In this context, M. Abou-Gharbia and co-workers [Contribution 4] offer an interesting perspective on the dual nature of dezocine as an opioid analgesic, suggesting that its advantages may outweigh its limitations. The authors also discuss the ongoing development of pharmaceutical formulations that enable alternative routes of administration to intravenous and intramuscular ones [32], which could pave the way for the sub-chronic and chronic use of dezocine.

Since opioids are routinely used to treat severe pain, and since sepsis is one of the most challenging complications in intensive care medicine, it is important to study the impact of opioid use on sepsis sequelae. M. M. El-Mas and colleagues [Contribution 5] present an original research article on the contribution of central inflammatory and oxidative stress pathways to the morphine-induced exacerbation of the cardiovascular effects of sepsis, using a cecal ligation and puncture septic rat model. Interestingly, by using selective inhibitors, this study demonstrated that morphine aggravates sepsis-induced cardiovascular and autonomic disturbances via µ-opioid receptor activation and the upregulation of central inflammatory, oxidative stress, and chemotactic signals. Using the same animal sepsis model, El-Mas previously reported that morphine accentuates decreased systolic blood pressure, as well as memory, learning, and behavioral alterations. He correlated these with intensified inflammatory and histopathological signs of hippocampal damage [33]. Morphine alone has been shown to induce hemodynamic changes in humans, such as impaired heart rate responses [34], altered cardiac output, decreased vascular resistance, and hypotension [35,36]. Furthermore, chronic morphine treatment has been shown to alter the gut microbiome, disseminate Gram-positive bacteria from the gut, and suppress endotoxin tolerance, leading to persistent inflammation, sepsis, and septic shock [37,38]. If these observations are translatable to humans, they underline the need to reconsider morphine use in patients with sepsis, and more generally in patients with cardiovascular dysfunction or autonomic instability.

Together, the articles in this Special Issue provide new insights into the cellular and molecular mechanisms of opioid toxicity and offer fresh perspectives on repurposing opioid drugs. While advances in receptor biology, metabolism, and drug delivery systems unravel promising new avenues, the rapid emergence of new synthetic opioids and their increasing misuse require urgent, tailored responses. Understanding dose–response dynamics and interindividual variability is essential for the development of safer and more effective opioid drugs. Furthermore, sustained progress requires not only scientific innovation but also intricate coordination between clinicians, researchers, regulatory bodies, and public health stakeholders. The complexity of toxicological outcomes evolves alongside opioid pharmacological properties, demanding a delicate balance between optimizing pain management and minimizing harm. When navigating this complexity, the ultimate goal of preserving the therapeutic potential of opioids while protecting individuals and communities from their most devastating consequences must never be disregarded.
